# Editorial: Ferroptosis in malignant brain tumors

**DOI:** 10.3389/fonc.2023.1276971

**Published:** 2023-09-29

**Authors:** Eduard Yakubov, Ali Ghoochani, Nicolai Savaskan

**Affiliations:** ^1^ Department of Neurosurgery, Paracelsus Medical University, Nuremberg, Germany; ^2^ Department of Chemical Engineering, Stanford University, Stanford, CA, United States; ^3^ Canary Center at Stanford for Cancer Early Detection, Stanford University, Stanford, CA, United States; ^4^ Department of Neurosurgery, University Medical School Hospital, Universitätsklinikum Erlangen (UKER), Friedrich–Alexander University Erlangen–Nürnberg (FAU), Erlangen, Germany; ^5^ Department of Public Health Neukölln, District Office Neukölln of Berlin Neukölln, Berlin, Germany

**Keywords:** ferroptosis, brain tumor, glioblastoma, drug resistance, tumor microenvironment, brain edema

Malignant primary brain tumors constitute around 30% of all primary brain tumor diagnoses in the United States. Unfortunately, these type of tumors still have a fatal prognosis despite advancements in the neuro-oncological toolbox. Although a multimodal therapy approach is the current gold standard, malignant primary brain tumors display a complex intratumoral heterogeneity. As a consequence, brain tumors trigger intricate molecular and metabolic shifts within the tumor microenvironment which might be responsible for therapy resistance and tumor relapse. However, our understanding of the molecular composition and orchestration of malignant primary brain tumors is still incomplete for efficient clinical translation. Thus, further investigations into the mechanisms of brain tumor growth are urgently needed.

Recent research has highlighted the relevance of ferroptosis in tumorigenesis – a process of iron-dependent programmed cell death. This novel mechanism also reveals clinical relevance due to its potential to mitigate oxidative stress and treatment resistance. However, the underlying mechanisms and regulators of ferroptosis remain elusive, with limited dedicated research. Consequently, triggering ferroptosis emerges as a promising therapeutic route, particularly for malignant brain tumors, which demand a new treatment paradigm.

This Research Topic aims to provide a comprehensive overview of ferroptosis in brain tumor development, progression, recurrence, and its interplay with the immune and tumor microenvironment, along with its therapeutic prospects. This endeavor culminated in a collection of five original research articles and twelve review articles, contributed by eminent global ferroptosis researchers.

Several manuscripts have illuminated distinct aspects of ferroptosis in malignant gliomas. Zhou et al. investigated the impact of miR-29b-mediated targeting of GPx7 (glutathione peroxidase 7), revealing that GPx7 suppression enhances erastin-induced ferroptosis. Dong et al. uncovered the influence of ferroptosis-related genes on immunity, stemness and prognosis in glioblastoma, suggesting novel prognostic indicators. Fu et al. identified LncRNA (long noncoding RNA) PELATON as a ferroptosis suppressor and prognostic signature, introducing fresh insights into the intricate molecular landscape of these tumors. Kram et al. showcased an upregulation of ACSL4 (acyl-CoA synthetase long-chain family member 4) and ALDH1A3 (aldehyde dehydrogenase 1A3) proteins during tumor relapses, indicating an increased vulnerability of glioblastoma relapses to ferroptosis. For further understanding of the dynamic progression and structural attributes of necrosis in glioblastomas, Yee et al. conducted a timely study of necrosis development in a mouse glioblastoma model, linking radiographic and histological observations. The extent of necrosis seen among glioblastoma patients was reconstructed using orthotopic xenograft glioma model induced by hyperactivation of the Hippo pathway transcriptional coactivator with PDZ-binding motif (TAZ).

Within the realm of review articles, Zhao et al. meticulously examined the pivotal role of iron transporters in ferroptosis within malignant brain tumors. This exploration seamlessly segued into the realm of PPARγ modulation in further research by the Yakubov team unraveling the intricate interplay between this molecular orchestrator and the ferroptosis process within the context of malignant glioma and tumor-related edema. On the other hand, Ferrada et al. embarked on an exploration of pharmacological avenues to incite ferroptosis, specifically targeting glioblastoma and neuroblastoma. Meanwhile, Chi et al. illuminated the potential benefits and challenges of harnessing ferroptosis in treatments, revealing insights into the distinctive molecular and microenvironmental traits inherent to these separate brain tumors. A detailed analysis of the immunological milieu of gliomas by Wang et al. identified ferroptosis’s role within the complex immune microenvironment.

The remaining publications in this Research Topic focus on the current situation, prospects, drug applications and off-target effects of ferroptosis induction in malignant brain tumors. Lu et al. provided insights into the molecular mechanisms of ferroptosis in glioma progression and treatment, while Yin et al. discussed the mechanisms of long non-coding RNAs in glioma ferroptosis. Zhang et al. explored ferroptosis-related ncRNAs in an effort to achieve personalized treatment regimen for gliomas through ferroptosis. Yao et al. proposed a ferroptosis-based drug delivery system for malignant brain tumors. Zhou et al. highlighted the emerging role of ferroptosis as a promising therapeutic target in glioblastoma treatment, particularly in cases that are resistant to conventional therapy. Xie et al. explored autophagy-dependent ferroptosis as a potential treatment for glioblastoma. In addition, Dahlmanns et al. analyzed genetic profiles of ferroptosis in malignant brain tumors and off-target effects of ferroptosis induction, emphasizing the need for precision in harnessing this therapeutic strategy.

Collectively, the collection of research in this Research Topic provides a comprehensive panorama of ferroptosis’s multifaceted involvement in malignant brain tumors. These articles accentuate specific molecular targets, such as GPx, long non-coding RNAs, iron transporters, and PPARγ, in the context of distinct brain tumor types. The diverse array of mechanisms, prospective therapeutic pathways, and challenges associated with ferroptosis in malignant brain tumors underscore the imperative need for further research to unlock its full therapeutic potential. This pursuit not only opens avenues for innovative strategies but also holds the potential to reshape the treatment terrain for malignant brain tumors, ushering in renewed optimism for both patients and the neuro-oncological community.

## Author contributions

EY: Supervision, Writing – original draft, Writing – review & editing. AG: Writing – review & editing. NS: Writing – review & editing.

